# Phytochemical analysis, antioxidant capacities, and in vitro biological activities of the extract of seed coat as by-products of pea

**DOI:** 10.1186/s13065-023-00911-8

**Published:** 2023-02-01

**Authors:** Hanaa S. S. Gazwi, Maha O. A. Omar, Magda E. Mahmoud

**Affiliations:** 1 Department of Agricultural Chemistry, Faculty of Agriculture, Minia University, El-Minya, Egypt; 2 Department of Microbiology, Faculty of Agriculture, Minia University, El-Minya, Egypt

**Keywords:** Seed coat pea, Antioxidant, Antimicrobial, Antiviral, Leukemia, Lymphoma

## Abstract

Converting seed coat peas (hulls) (SCP) into beneficial products provides a solution for waste treatment. This study aimed to investigate the phytoconstituents and biological activities of SCP extract. Phytochemical screening, total bioactive compounds, and GC–MS analysis were evaluated. Then, the antioxidant, antibacterial, anticancer, and antiviral activities of SCP extract were determined. The results demonstrate that SCP extract has bioactive compounds such as carbohydrates (29.53 ± 4.23 mg/mL), protein (0.24 ± 0.02 mg/mL), phenolics (27.04 ± 0.94 mg GAE/g extract), and flavonoids (17.19 ± 0.48 mg QE/g extract). The existence of more than 16 substances was determined using GC–MS analysis. The extract showed potential antioxidant activities, with the maximum activity seen for extract (IC50 µg/mL) = 79.16 ± 1.77 for DPPH, 67.40 ± 5.20 for ORAC, and 61.22 ± 4.81 for ABTS assays. The SCP extract showed potent antimicrobial activity against four gram-positive bacteria (*Bacillus cereus*, *Streptomyces sp*., *Staphylococcus aureus*, and *Salmonella sp*.) and two gram-negative bacteria (*Escherichia coli* and *Pseudomonas sp*.). SCP extract exhibited potential anticancer activity against lymphoma U937 and leukemic cells (THP1). The extract exhibited potential antiviral activity, with a selectivity index (SI) equal to 11.30 and 18.40 against herpes simplex-II (HSV-2) and adenovirus (Ad7), respectively. The results demonstrate more accurate information about peas by-products' chemical and antioxidant activities in various applications. The chemical components of peas by-products were found to have an in vitro antioxidant, antibacterial, and antiviral activity against leukemia and lymphoma.

## Introduction

The widespread use of antibiotic-resistant bacteria is a major public health concern [1]; this is mostly the consequence of deliberate efforts to genetically modify germs to be resistant to a certain medicine. Meanwhile, studies have shown that viruses are among earth's most common forms of life [2, 3]. Millions of individuals are sick with viruses, and many do not get treatments or vaccinations, particularly for retrovirus infections [4]. Infections with the hepatitis C virus (HCV), adenovirus type 7 (ADV7), or herpes simplex virus type 1 (HSV1) are universally devastating illnesses that may cause a variety of liver complications, as well as mortality and genital herpes [5]. Constant effort is put towards discovering a more effective material derived from nature that may mitigate this threat to public health. The potential of several plants as antibacterial and antiviral agents has been studied [6].

One study found that lymphoma accounts for 3% of all cancers globally [7]. Since lymphoma is linked to immune system damage, some hereditary illnesses, immunosuppressive medication regimens, and specific viruses are recognized as risk factors [7]. Myeloid blood cells proliferate too quickly and unisexually, giving rise to the hematological malignancy known as acute myeloid leukemia (AML) [8]. About 351,000 new instances of leukemia are diagnosed annually across the globe, accounting for 2.8% of all cancers and 3.4% of all cancer deaths [9]. Clinical therapies such as bone marrow transplant, radiation, and chemotherapy are used to treat leukemia patients; however, most treatments are not yet suitable due to the significant side effects that are still encountered. Because of their ability to prevent tumor development at several stages, phytochemicals are gaining significance as a source [10].

The pea (*Pisum sativum* L.) is a nutritious leguminous crop that is extensively farmed [11]. Pea has a good balance of macronutrients, like dietary fibers, carbohydrates, high-quality proteins, minerals, and vitamins, and is good for health [12]. Peas also have a lot of antioxidants, which mostly come in the form of phenols, carotenoids, and tocopherols [12]. Legume seed coats are natural antioxidants with anti-inflammatory, heart-protective, anticancer, and other beneficial properties that work with protein-rich seeds to improve health [13]. Pea seed coats have a lot of bioactive phenolic compounds. Moreover, it has a lot of flavonoid compounds, which are becoming known for their ability to fight free radicals and act as antioxidants [14].

On the other hand, the waste products of the peas industry processing must be considered to avoid pollution. As a result, it is vital to examine their chemical composition and biological activities to maintain appropriate usage of these wastes in various applications [15]. Therefore, this study is the first to validate the phytochemical analysis, antioxidant capacities, and in vitro biological activities of the extract of SCP.

## Methodology

### SCP prepared extract

SCP was acquired from the vegetable processing sector in El-Minya, Egypt, and identified by professor Raga A. Taha, Horticulture Department, Faculty of Agriculture, Minia University. These samples were dried, blended, homogenized by grinding to a fine powder, then passed through a 1 mm sieve. SCP powder was extracted by water (1:10 w/v) after three hours of stirring at room temperature (25 °C). Whatman No. 1 filter paper was used for filtration. Then, the filtrate was filtered again to maximize the extract. The solution was filtered and dried in an oven at a temperature of 45–50 °C. The extract was kept at 4 °C for future use. The collection of SCP complies with relevant institutional, national, and international guidelines.

### Phytochemical screening

The major phytochemical compounds of the extract were tested qualitatively, according to Jamil et al. [16]. Alkaloids, flavonoids, glycosides, tannins, saponins, terpenes, and phenolics were among the significant studied components.

### Bioactive compounds

#### Total soluble carbohydrate content

Total soluble carbohydrate analysis was performed using the method of Gerhardt et al. [17]. The total soluble carbohydrates of the samples are presented as µg glucose/mg sample.

#### Total protein content

10 mg of sample was dissolved in 10 mL phosphate buffer saline pH 7.4. The sample was sonicated for 30 min. The sample was then filtrated using a 0.45um syringe filter. According to the manufacturer's instructions, the BCA protein assay kit (Novagen) was used to test the sample.

#### Total phenolic content (TPC) and total flavonoid content (TFC)

The TPC of the extract was defined spectrophotometrically according to the Folin-Ciocalteu colorimetric technique reported by Haq et al. [18]. The sample results are presented as µg Gallic acid equivalent/mg extract. The TFC of the extract was measured by the aluminum chloride colorimetric technique [19]. The sample results are presented as µg quercetin equivalent/mg extract.

### Gas chromatography–mass spectrometry (GC–MS) analysis

The GC–MS analysis of the SCP extract was performed according to a previously described method [20]. GC–MS was conducted at the National Center Dokki's mass spectrometry laboratory in Giza, Egypt, using a Thermo Scientific Trace GC Ultra ISQ single Quadrupole MS, TG-5MS fused silica and capillary column (30 m × 0.25 mm × 0.25 μm film thickness). The column oven was set to 50 °C, then 200 °C at 7 °C/min, held for 2 min, then 290 °C, increased at 15 °C/min, and held for 2 min. After a 4-min solvent delay, an AS3000 autosampler and GC in the split mode were employed to inject 1 μL of the diluted sample automatically. An electron ionization device with an ionization energy of 70 eV was used. Helium gas was used as the carrier gas and flowed at a constant rate of 1 mL/min. The injector and the MS transfer line were adjusted to 270 °C and 250 °C, respectively. The percent relative peak area was employed to test the possibility of quantifying the components.

### Evaluation of the in vitro biological activities

#### In vitro antioxidant activity

Three complimentary in vitro assays were applied to determine the antioxidant activity of the SCP extract: DPPH-free radical scavenging, the oxygen radical absorbance capacity (ORAC), and ABTS + radical cation scavenging. Lu et al. [21] technique was utilized to measure DPPH, and the Hao et al. [22] method was used to assess the ability to neutralize the radical ABTS+. The ORAC test was conducted using the technique of Liang et al. [23].

#### Antibacterial activity

Antibacterial activity of SCP extract against four gram-positive bacteria (*Bacillus cereus*, *Staphylococcus aureus*, *Streptomyces *sp., and* Salmonella *sp.) and two gram-negative bacteria (*Escherichia coli* and *Pseudomonas *sp.) was evaluated using an optimized method by Manoj et al. [24]. Three concentrations of SCP extract were prepared (40, 60, and 80%). A single colony of each bacterium was transferred into a 5 mL nutrient broth medium. The bacterial inoculums were adjusted to 10^6^ CFU (Colony Forming Units)/mL. One milliliter of each bacterial inoculum was poured with 10 mL of nutrient agar medium [25] into a 10 cm Petri plate. Five-millimeter diameter sterile filter paper discs were saturated with SCP extract concentrations. Sterilized water was used for control discs. Four paper discs were placed into each Petri dish, one for control and three representing the three concentrations. The plates were incubated at 37 °C for 24 h. Following incubation, the diameter of each growth inhibition zone was measured in mm. Three replicates were used for each treatment, and the experiment was repeated twice.

#### Antiviral activity

Nawah-Scientific, Egypt, provided the Herpes simplex virus type II and Vero cells. Vero cells were cultured in DMEM media containing 10% fetal bovine serum and 0.1% antibiotic/antimycotic solution. The trypsin–EDTA antibiotic, antimycotic solution, DMEM medium, and fetal bovine serum were provided by Gibco BRL (Grand Island, NY, USA). The crystal violet method evaluated the antiviral activity and cytotoxicity assays using the recently reported cytopathic (CPE) inhibition effect [26]. In brief, Vero cells were seeded at a density of 2 × 10^5^ cells/well into a 96-well plate one day before infection. The following day, the growth media were withdrawn, and the cells were rinsed with phosphate-buffered saline.

The virus's infectivity was measured by the crystal violet technique, which monitored CPE and allowed for calculating the proportion of viable cells. A 0.1 mL diluted virus suspension containing 50% of the virus stock's CCID50 (cell culture infective dose) was introduced to mammalian cells. Three days after infection, this dose was chosen to produce the necessary CPEs. The 0.01 mL of media containing the required compound concentration was used to treat the cells. Then, the antiviral activity of each test sample was tested using a tenfold diluted concentration range of 0.1–10,000 g/mL. The cell controls (nondrug-treated, non-infected cells) and the viral controls (nondrug-treated, virus-infected cells). Culture plates were incubated at 37 °C in 5% CO_2_ for 72 h. Light microscopy was used to monitor the development of the cytopathic effect. After washing with PBS, the monolayers of cells were fixed and stained with a 0.03% crystal violet solution in 10% formalin and 2% ethanol. After washing and drying, individual wells' optical densities were evaluated spectrophotometrically at 570/630 nm. This equation determined the proportion of antiviral activity of the test compounds: antiviral activity = [(mean optical density of cell controls-mean optical density of virus controls)/(optical density of the test-mean optical density of virus controls) times 100%. Based on these findings, the 50% CPE inhibitory dosage (IC_50_) was established. We performed a cytotoxicity assay before this assay. Cells were seeded at 2 × 10^4^ cells/well density in a 96-well plate. After that, the cells were reintroduced to a culture medium containing serially diluted samples and cultured for 72 h. Then, the media were withdrawn, and the cells were rinsed with PBS. The following procedure was performed identically to those reported before for the antiviral activity assay.

### SCP extract against leukemia (THP1) and lymphoma (U937)

#### Cell culture

Nawah Scientific Inc. provided THP1: acute monocytic leukemia (AML) and U937: human lymphoma (Mokatam, Cairo, Egypt). At 37 °C, cells were cultured in RPMI medium containing 10% heat-inactivated fetal bovine serum, 100 units/mL penicillin, and 100 mg/mL streptomycin.

#### Cytotoxicity assay

The WST-1 test was used to determine the viability of cells using an Abcam^®^ kit (ab155902 WST-1 Cell Proliferation Reagent). Aliquots of 50 L cell suspension (3 × 10^3^ cells) were seeded in 96-well plates and cultured for 24 h in complete media. Cells were then treated with another portion of 50 μL media with serial doses of drugs. After 48 h of drug exposure, cells were treated with a 10 μL WST-1 reagent, and the absorbance at 450 nm was determined following 1 h using a BMG LABTECH^®^-FLUOstar Omega microplate reader (Allmendgrün, Ortenberg).

### Statistical analyses

The chemical assays were performed in triplicate, and the results were demonstrated as the mean and standard deviation. One-way ANOVA was used to carry out the statistical analysis. Furthermore, the LSD test was used to examine the statistical significance of mean differences, with a p-value equal to 0.05. GraphPad Prism was used to conduct the statistical analysis.

## Results and discussion

### Phytochemical analysis

The presence of therapeutically active phytochemicals alkaloids, flavonoids, glycosides, tannins, phenolic, terpenes, and the absence of saponins was discovered during a phytochemical screening of SCP, as shown in Table 1.

**Table 1:** Phytochemical analysis of SCP extract

Phytochemical	Results
Alkaloids	** + **
Flavonoid	** + **
Glycosides	** + **
Tannins	** + **
Saponins	−
Terpenes	−
phenolic	** + **

− Absent, + Present

These phytoconstituents (flavonoids, alkaloids, glycosides, tannins, and phenolic substances) may be responsible for the health benefits of the extract of SCP. The alkaloids are employed as analgesics, hallucinogens, stimulants, anesthetics, and antibacterial agents, and glycosides are said to have potent antibacterial properties [27]. Tannins are plant metabolites with antibacterial capabilities, and phenolics have an antioxidant capacity, which may help the body fight pathology-induced free radical production [28].

The existence of phenolic compounds in peas’ seed coats was verified by Duenas et al. [29]. Pea was analyzed for their phenolic profiles and antioxidant capacities by Troszynska and Ciska [11]. Free and esterified phenolic acids were detected in pea, with the latter having greater quantities in the seed coat. The seed coat contained condensed tannins, which have been demonstrated to have extremely significant antioxidant activity [30].

### Total bioactive compounds

Total proteins, carbohydrates, flavonoids, and phenols were evaluated to assess total bioactive content. The SCP extract contained a substantial amount of proteins (0.24 ± 0.02 mg/mL extract), Carbohydrates (29.53 ± 4.23 mg/g), flavonoids (15.89 ± 0.28 mg QE/g extract), and phenolic (22.37 ± 0.59 mg GAE / g extract) as shown in (Table 2).

**Table 2:** Total bioactive compounds in SCP extract

	Total Phenolic Content (mg GAE/g extract)	Total Flavonoid Content (mg QE/g extract)	Total Protein Content (mg/g extract)	Total soluble carbohydrate (mg/g extract)
SCP extract	27.04 ± 0.94	17.19 ± 0.48	0.24 ± 0.02	29.53 ± 4.23

All results are expressed as Mean ± SD (n = 6)

To our knowledge, there is little or no available data in the literature on the total proteins, carbohydrates, flavonoids, and phenolics of the studied extract. Comparing our findings to Oomah et al. [31] revealed a total phenolic content of yellow pea hulls ranging between 2.6 and 9.1 mg catechin/g material, considering the extraction solvent. The phenolic content of black soybean seed coats from 60 categories ranged between 0.512 and 60.58 mg GAE/g [32]. The total phenolic content of lentil seed coats from various types ranged from 24.63 to 87.16 mg of catechin equivalents per gram of dry weight [31]. As a result, the total phenolic content values of the seed coats of the peas studied are equivalent to those of other widely farmed pulse crops such as lentils or soybeans. Several biological properties of phenolics have been reported, especially the significant antioxidant activity mentioned by Cao et al. [33] correlating with this study's findings. The extract showed a high percentage of protein, which increased its nutritional value.

### GC–MS analysis of the extract

**Fig. 1:**
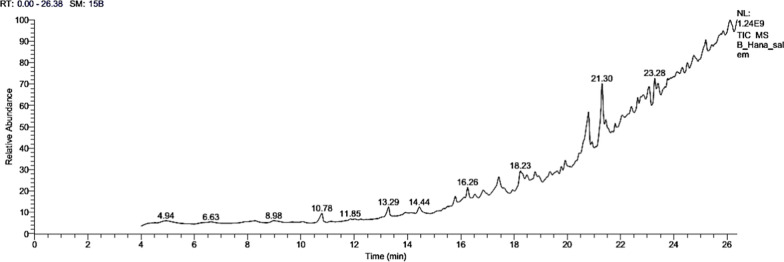
GC–MS of Chromatogram of SCP extract

**Fig. 2:**
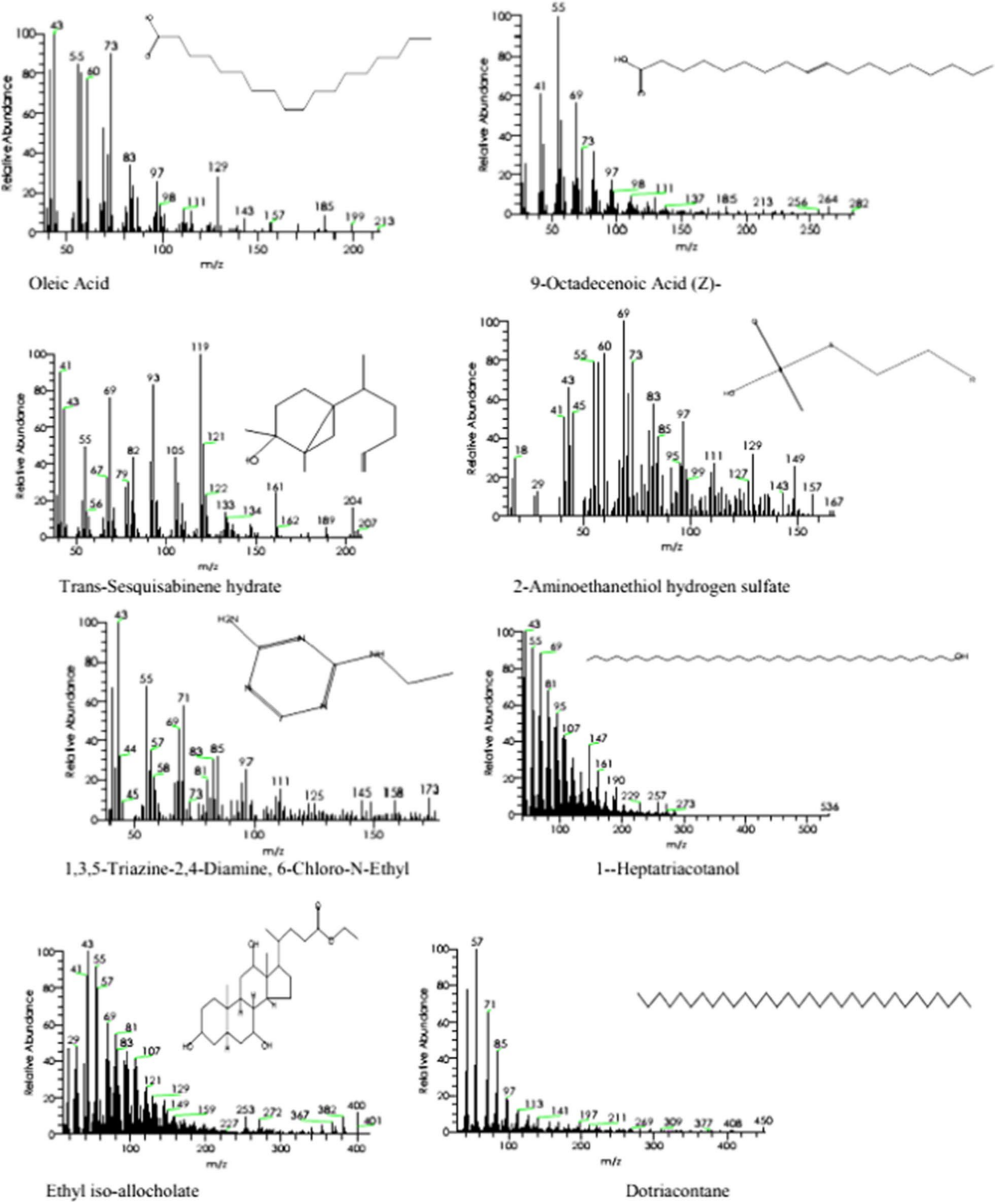
Mass spectrums molecular structures of components

**Fig. 3:**
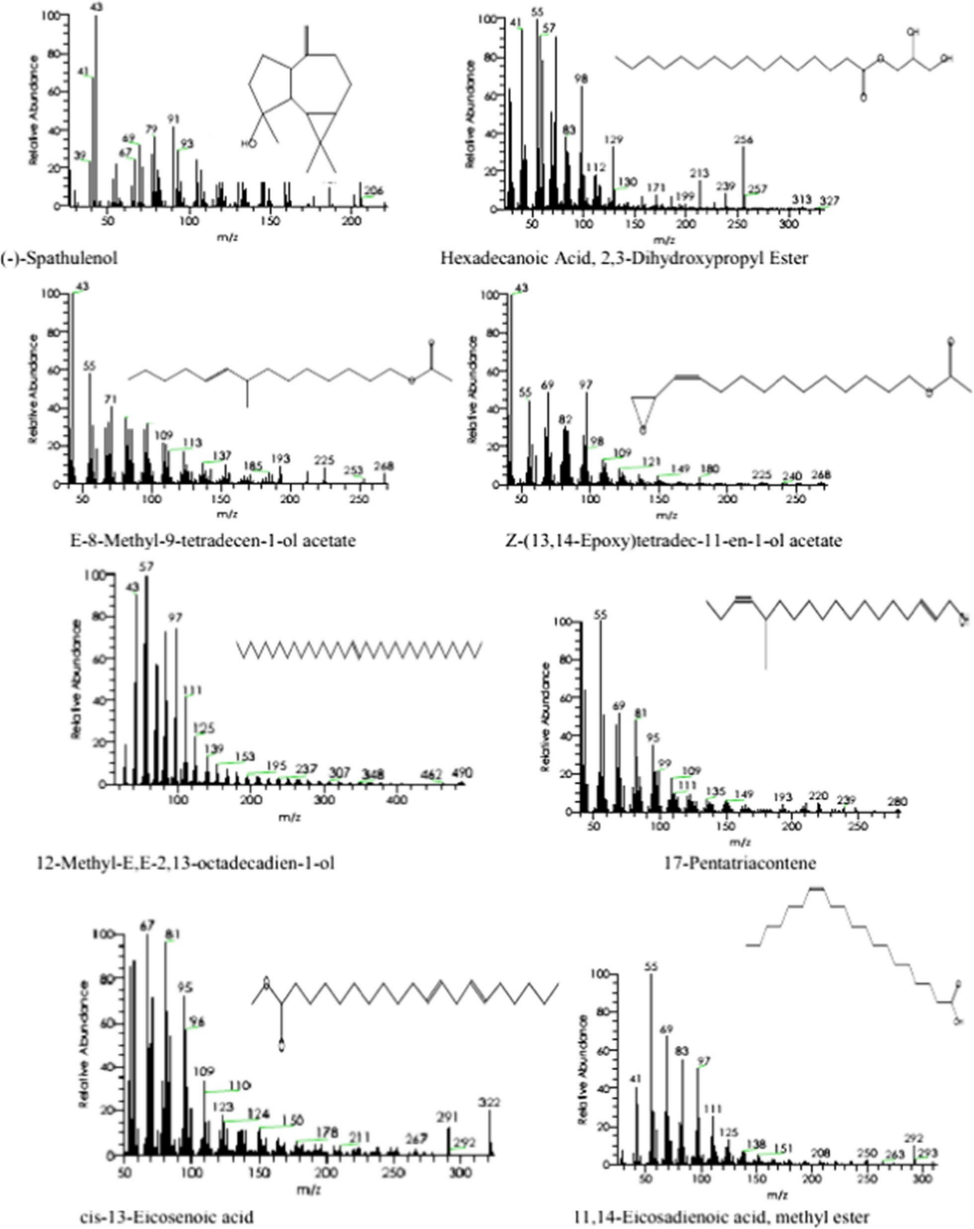
Mass spectrums molecular structures of components

Sixteen compounds in the SCP extract were identified using GC–MS, as shown in Table 3. The major constituents are (-)-Spathulenol (14.55%), Hexadecanoic acid, 2,3-Dihydroxypropyl Ester (10.86%), 12-Methyl-E, E-2,13-octadecadienoic-1-ol (6.57%), 11,14-Eicosadienoic acid, methyl ester (6.61%), cis-13-Eicosenoic acid (5.78%) (Table 3, Fig. 1). The components' fragmentation is shown in (Figs. 2 and 3).

**Table 3:** GC–MS analysis of SCP extract

No.	RT	Name of the compound	MF	MW	Peak area (%)	Compound nature	Activity
1	10.79	9-Octadecenoic Acid (Z)-	C_18_H_34_O_2_	282	2.61	Fatty acids	Antihypertensive increases HDL, and decreases LDL [35]
2	13.29	Oleic Acid	C_18_H_34_O_2_	282	2.72	Fatty acids	Antimicrobial [36], hypercholesterolemic [29], dermatitigenic [37], anti-inflammatory, and anti-tumor activity [38]
3	15.8	2-Aminoethanethiol Hydrogen Sulfate	C_2_H_7_NO_3_S_2_	157	2.25	Ester	No activity reported
4	16.25	Trans-Sesquisabinene hydrate	C_15_H_26_O	222	2.93	Terpenoids	Anti-cancer [39]
5	17.42	1-Heptatriacotanol	C_37_H_76_O	536	3.41	Alcohol	Antimicrobial activity [40]
6	18.23	1,3,5-Triazine-2,4-Diamine, 6-Chloro-N-Ethyl	C_5_H_8_ClN_5_	173	5.25	1,3,5-triazine-2,4-diamines	No activity reported
7	18.79	Dotriacontane	C_32_H_66_	450	2.48	Alkanes	Antimicrobial, antioxidant, antispasmodic, antibacterial, and antiviral [41]
8	19.92	Ethyl iso-allocholate	C_26_H_44_O_5_	436	3.03	Steroid	Antimicrobial activity, and anti-inflammatory [42]
9	20.8	Hexadecanoic Acid, 2,3-Dihydroxypropyl Ester	C_19_H_38_O_4_	330	10.86	Ester	Antimicrobial activity, and anticancer activity on cancer cell lines [43]
10	21.3	(-)-Spathulenol	C_15_H_24_O	220	14.55	Terpenes	Immunoinhibitory, anti-inflammatory, anticancer, and apoptosis inducer [44]
11	21.45	Z-(13,14-Epoxy) tetradec-11-en-1-ol	C_16_H_28_O_3_	268	1.54	Terpenes	Antioxidant, and hemolytic
		acetate					[45]
12	21.79	E-8-Methyl-9-tetradecen-1-ol acetate	C_17_H_32_O_2_	268	1.76	Acetate	No activity reported)
13	23.28	12-Methyl-E,E-2,13-octadecadien-1-ol	C_19_H_36_O	280	6.57	Alcohol	Antihistamine, antioxidant, analgesic, anesthetic, allergic, antibacterial, anticonvulsant, anti-salmonella,and antiseptic [46]
14	24.5	17-Pentatriacontene	C_35_H_7_0	490	2.29	Unsaturated aliphatic hydrocarbons	Anti-inflammatory, anticancer, antibacterial, and antiarthritic [47]
15	25.19	Cis-13-Eicosenoic acid	C_20_H_38_O_2_	310	5.78	Fatty acids	Anti-inflammatory activity [48]
16	26.11	11,14-Eicosadienoic acid, methyl ester	C_21_H_38_O_2_	322	6.61	Esters	Antimicrobial activity [49]

RT, relation time; MF, molecular formula; MW, molecular weight

These compounds have all been shown to have anticancer, anti-inflammatory, antioxidant, antimicrobial, antihypertensive, hypercholesterolemic, dermatitigenic, anti-tumor, antispasmodic, antiviral, immunoinhibitory, apoptosis-inducing, hemolytic, antihistamine, analgesic, anesthetic, allergic, anticonvulsant, anti-salmonella, antiseptic, and antiarthritic activity. Antioxidant activity is shown by Z-(13,14-Epoxy) tetradec-11-en-1-ol acetate, 12-Methyl-E, E-2,13-octadecadienoic-1-ol, and Dotriacontane. Antimicrobial activity is demonstrated by 12-Methyl-E, E-2,13-octadecadienoic-1-ol; 1-Heptatriacotanol; Dotriacontane; Ethyliso-allocholate; Hexadecanoic acid, 2,3-Dihydroxypropyl ester; 11,14-Eicosadienoic acid, methyl ester; 17-Pentatriacontene and Oleic acid. Anticancer activity is demonstrated by Trans-sesquisabinene hydrate, Oleic acid; 17-Pentatriacontene; (-)-Spathulenol; and Hexadecanoic acid, 2,3-Dihydroxypropyl ester. Anti-inflammatory activity is demonstrated by Oleic acid; cis-13-Eicosenoic acid; 17-Pentatriacontene, and (-)-Spathulenol. Correlations between phytochemicals and their biological activities are becoming more prevalent [34].

We describe the presence of biological activity of several significant components identified using GC–MS analysis. Thus, this form of GC–MS analysis is the first step toward elucidating the nature of the active components in peas. This type of study will aid in developing a more detailed study.

### Antioxidant activity

**Fig. 4:**
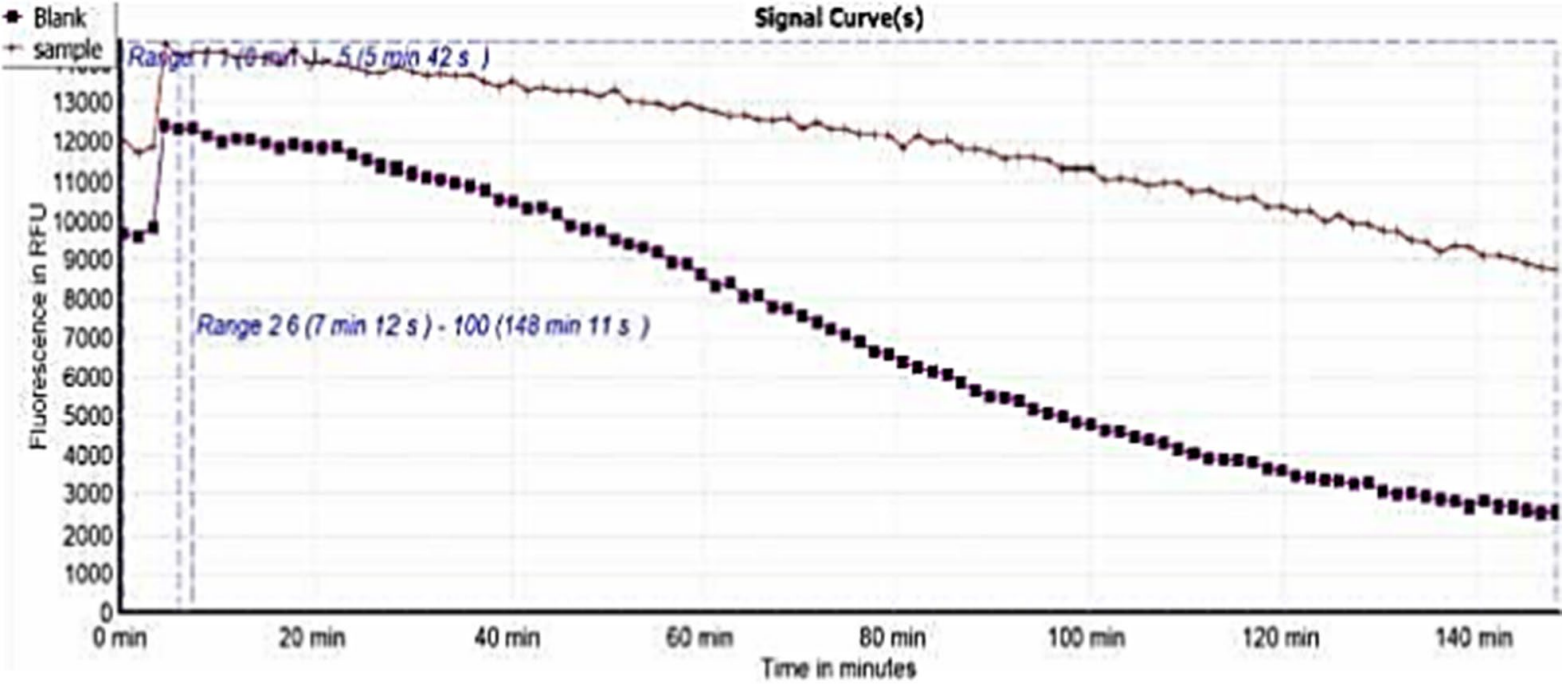
Signal curves indicating the decay of fluorescein upon applying the extract

Antioxidants are bioactive compounds with an inhibitory effect that reduces the damage produced by reactive free radicals. Compared to the standard Trolox, the outcomes of the antioxidant property evaluated by the three assessment methods, ABTS radical cation scavenging, DPPH radical scavenging, and ORAC tests, are shown in Table 4. According to the results, SCP extract showed higher antioxidant activity. In the ORAC test, the extract caused the fluorescence signal to decay (Fig. 4).

**Table 4:** Antioxidant activity of SCP extract

	DPPH (IC_50_ μg/mL)	ABTS (µM TE/mg extract)	ORAC (µM TE/mg extract)
SCP extract	79.16^a^ ± 1.77	61.22^a^ ± 4.81	67.40^a^ ± 5.20
Trolox	24.00 ± 0.16	35.30 ± 0.30	31.71 ± 0.01

All results are expressed as mean SD (n = 6)

^a^Significantly different from Trolox (TE) at (p ≤ 0.05)

Polyphenols found in vegetables are responsible for various biological reactions, including antioxidant activity [50]. According to Troszynska and Ciska [11], legume antioxidant compounds were primarily found in the seed coat. These findings are consistent with earlier research [51]. The seed coat, in general, plays a significant part in the chemical and physical defense mechanism of seeds exposed to oxidative damage, UV light, and other environmental conditions. As a result, the seed coat contains various bioactive substances, including polyphenols, which protect against oxidative stress [52].

### Antimicrobial activities

Infectious diseases are one of the world’s most significant challenges, with about 57 million people dying yearly [53]. Pharmacological firms have developed several novel antibiotics in the last three decades. However, their harmful effects and the widespread growth of multi-drug resistance (MDR) microorganisms reduce their effectiveness [54]. Therefore, the MDR efflux pump is crucial to finding novel and innovative treatments.

**Fig. 5:**
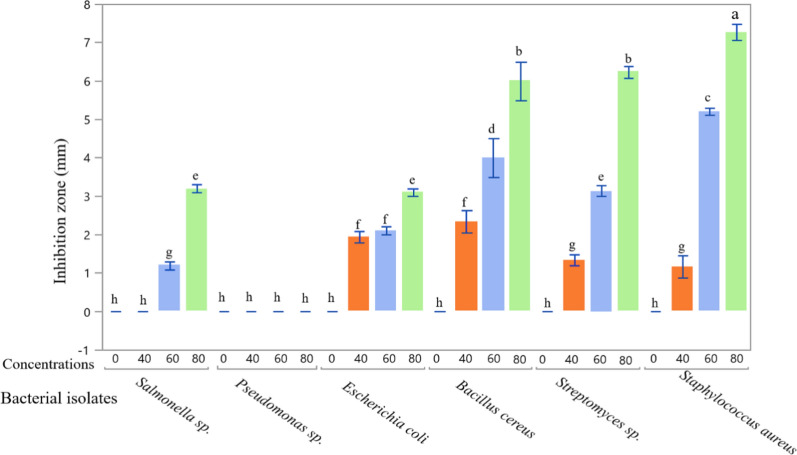
The impact of SCP extract at different concentrations on six bacterial strains. The data values were expressed as mean ± SD in mm of the inhibition zone demonstrated. All values with the same alphabetic superscript indicate no statistically significant differences, while those with different alphabetic superscripts indicate statistically significant differences according to Tukey's post-hoc analysis of variance (ANOVA)

**Fig. 6:**
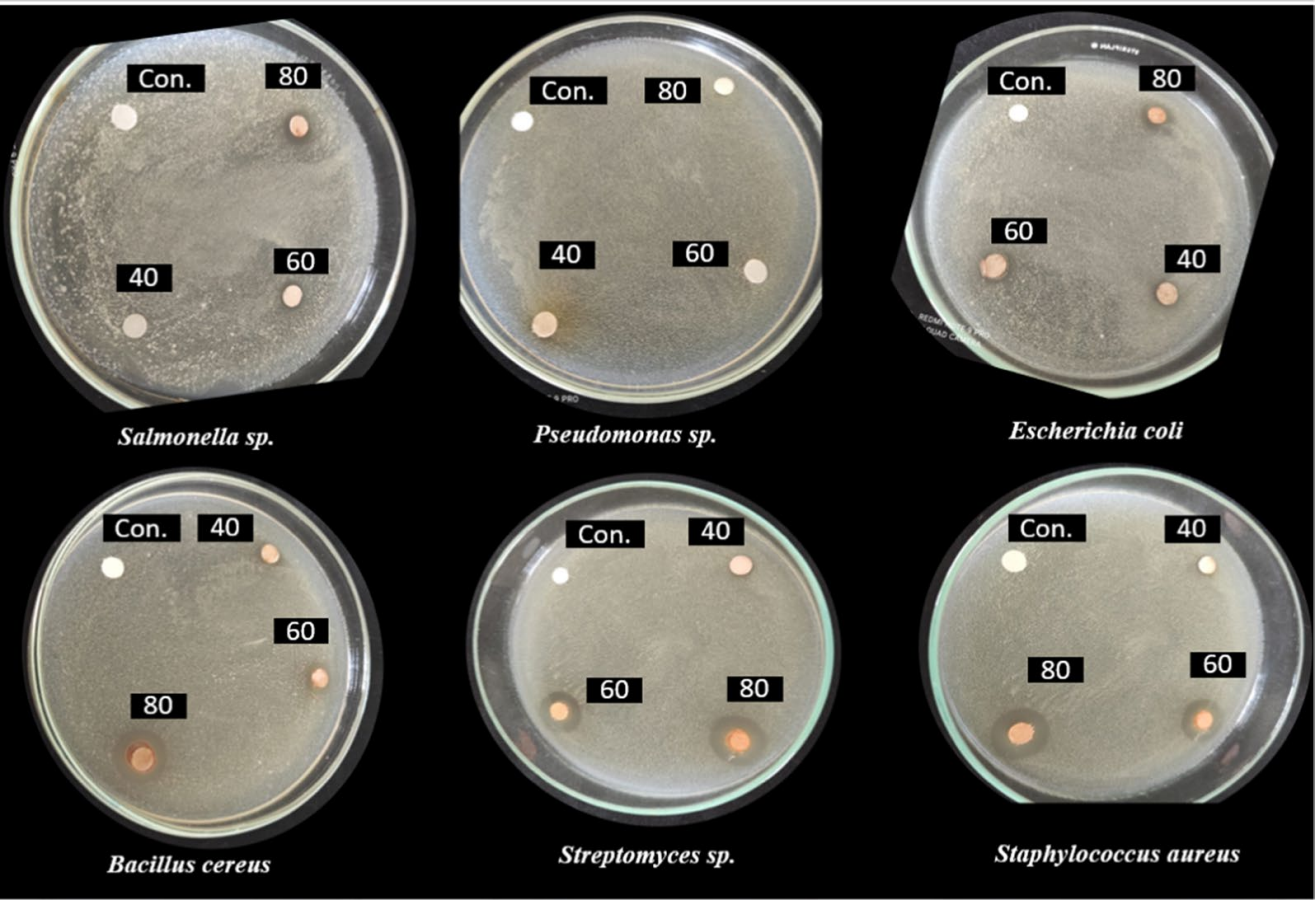
The interaction between SCP extract at different concentrations and six bacterial strains

Medicinal plants have improved patients' lives for thousands of years because they contain many chemical components that have a specific physiological effect on the human body. Alkaloids, flavonoids, tannins, saponins, terpenoids, and phenolics represent the most prominent chemicals. They have provoked the interest of pharmacists due to their medicinal efficacy and minimal toxicity [55]. SCP extract had various degrees of antibacterial activity (Figs. 5 and 6). SCP extract significantly affected all tested bacterial strains except for *Pseudomonas *sp. See Figs. 5 and 6. The effect of the three concentrations varied among the strains. In general, 80% SCP extract concentration has shown the highest inhibition zones compared with 40 and 60% control. Also, the obtained data revealed that the most affected bacterial strains belonged to gram-negative bacteria (*E. coli*, *Salmonella *sp., and *Pseudomonas *sp.). *Staphylococcus aureus* was the most sensitive strain to SCP extract, demonstrating a 7.27 mm inhibition zone at 80% concentration. *Streptomyces *sp. and *Bacillus cereus *at the same concentration of 6.23 and 6.0 mm, respectively.

Due to active chemicals that can reduce bacterial growth without necessarily getting into the bacterial cell, the extract could inhibit the above-listed gram-negative bacteria [56]. We discovered alkaloids and flavonoids in the extract have antibacterial properties. As a result, we conclude that these metabolites are responsible for their antibacterial properties. The results are consistent with Jaberian et al. [57] findings that plant extracts exhibit antibacterial properties, which they attribute to powerful chemicals such as flavonoids, alkaloids, tannins, and other substances. Tannins are potent detoxifying agents that prevent protein development by precipitating the protein components.

### Antiviral activity

**Fig. 7:**
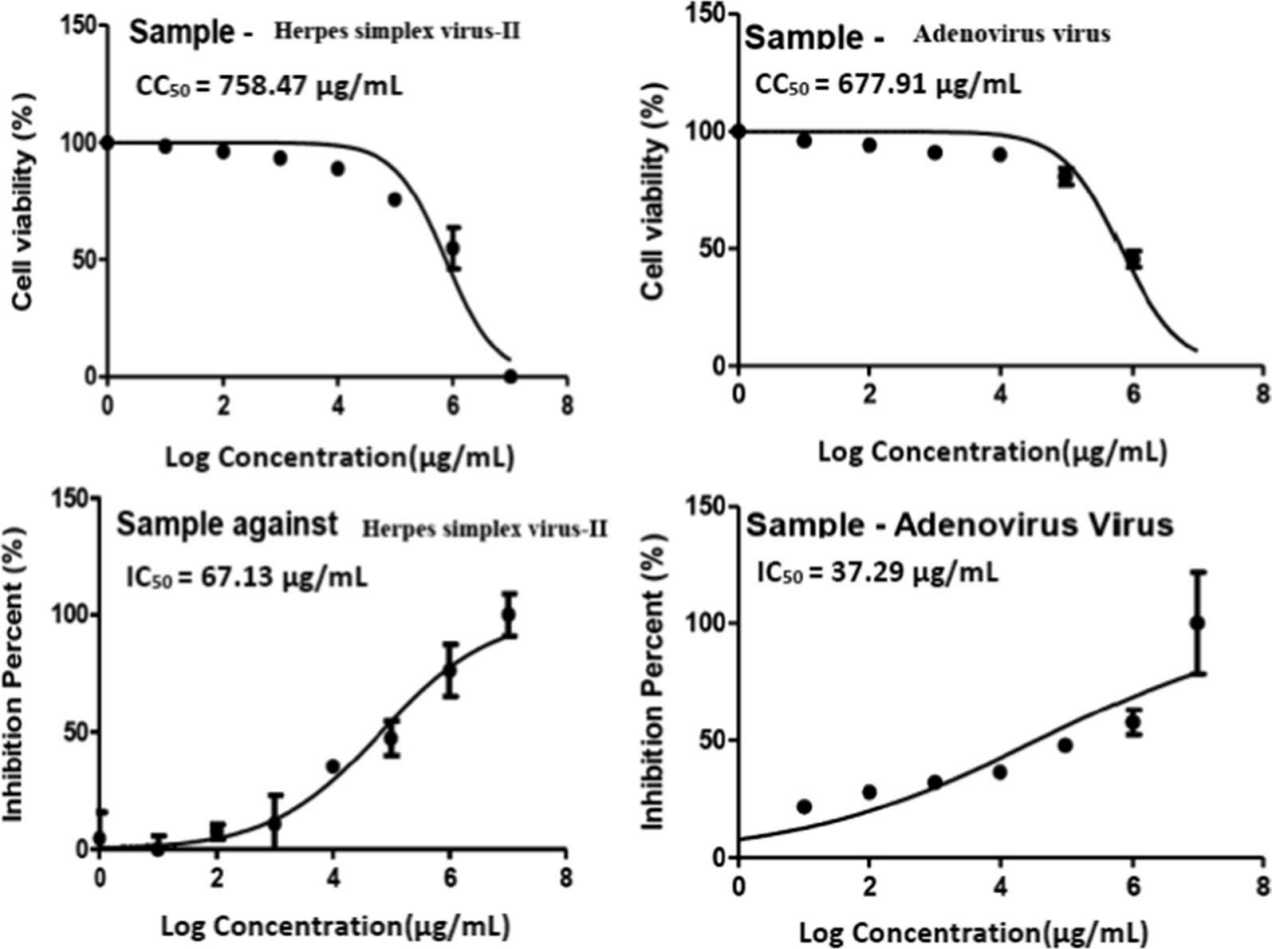
Cytotoxicity concentration 50 (CC_50_) and the 50% inhibitory concentration (IC_50_ (on Vero cells and HSV-2

Antiviral activity was tested against herpes simplex II and adenovirus viruses. The CPE-inhibition assay will identify potential antivirals against human Herpes simplex virus type II. The dose–response assay was designed to determine the range of efficacy for the chosen antiviral, i.e., the 50% inhibitory concentration (IC_50_) and the range of cytotoxicity (CC_50_). This assay is critical for determining antiviral efficacy in cell culture systems. The results of the 50% cytotoxic concentrations (CC_50_) and the 50% inhibitory concentration (IC_50_) were determined using GraphPad PRISM software (Graph-Pad Software, San Diego, USA). Table 5 shows that the SCP extract had strong antiviral activity against adenovirus, with a selectivity index of 18.2. Figure 7 shows the dose–response curves. The results in Table 5 show that the tested sample showed moderate antiviral activity against Herpes virus type-2 with a selective index = estimated CC_50_/estimated IC_50_ = 11.3. Consequently, the tested sample is a good candidate for further experiments as anti-Herpes viruses.

**Table 5:** Antiviral effect of SCP extract

	Virus	CC_50_ (µg/mL)	IC_50_ (µg/mL)	SI
SCP extract	Adenovirus	677.91	37.29	18.20
	Herpes simplex virus-II	758.47	67.13	11.30

SI, selectivity index = CC_50_/IC_50_; IC_50_, half-maximal inhibitory concentration; CC_50_, half-maximal cytotoxic concentration

The extract's antiviral impact seems mostly attributable to interacting with viral uptake, enzyme activity, replication, potentiating the host immune system, and functional peptides [58]. The anti-HIV action of the extract of SCP could be due to ubiquitin-like protein and lectin suppression of the reverse transcriptase enzyme, respectively [59]. Flavonoids treat HIV-1 protease and HIV-1 integrase enzymes in HIV replication [60].

### Leukemia and lymphoma cell lines

**Fig. 8:**
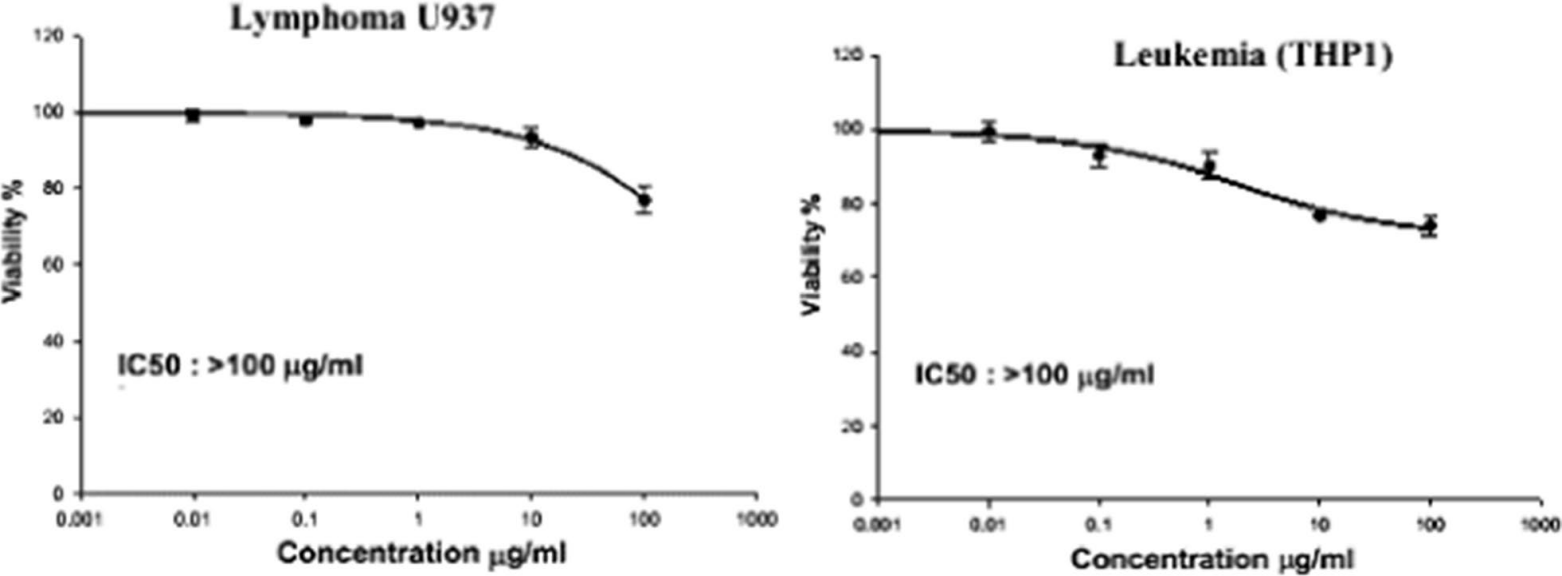
Cytotoxic effect of the SCP extract against lymphoma U937 and leukemic cells (THP1)

**Fig. 9:**
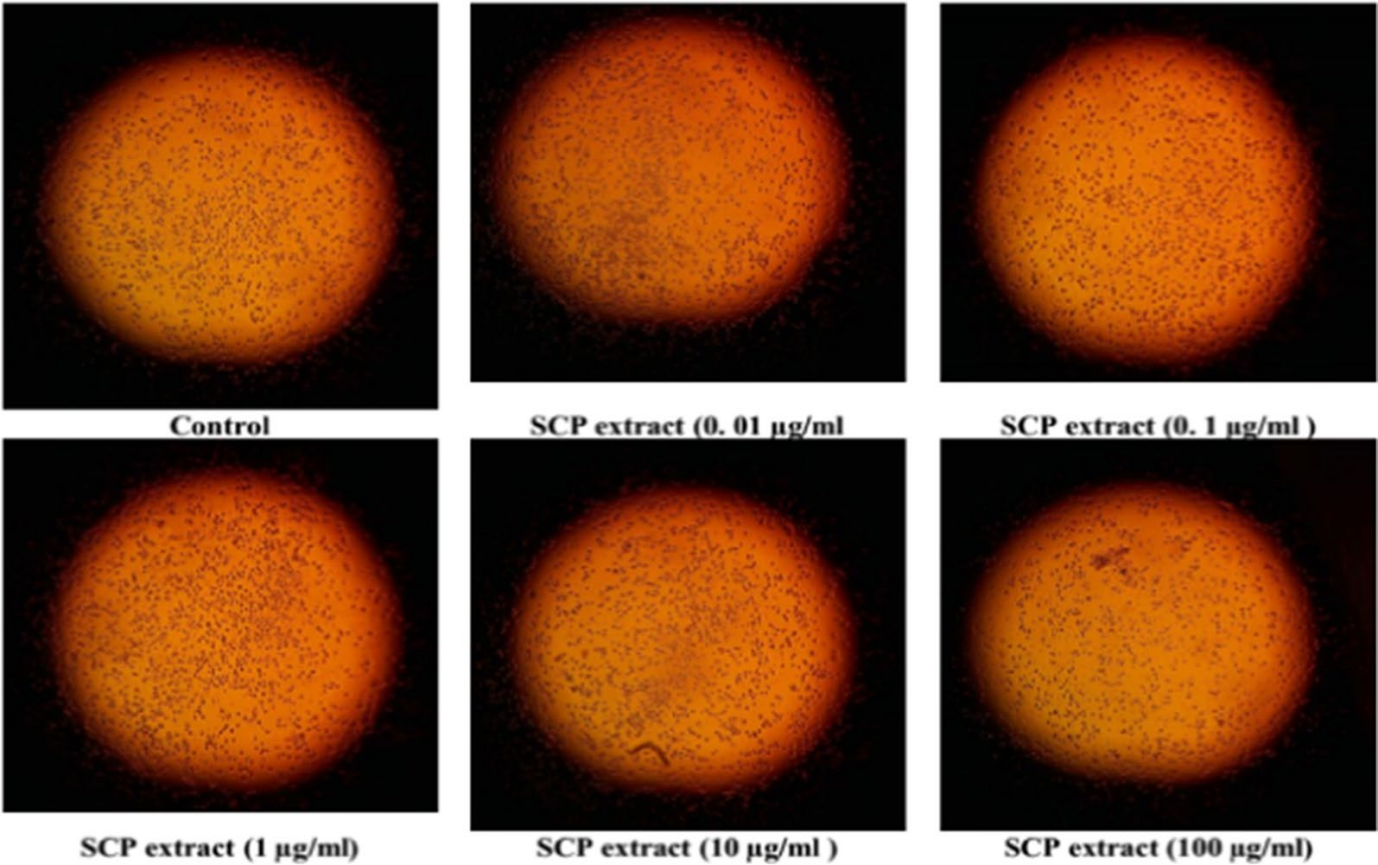
Photomicrographs of lymphoma U937cell lines for treatment with extract of SCP

**Fig. 10:**
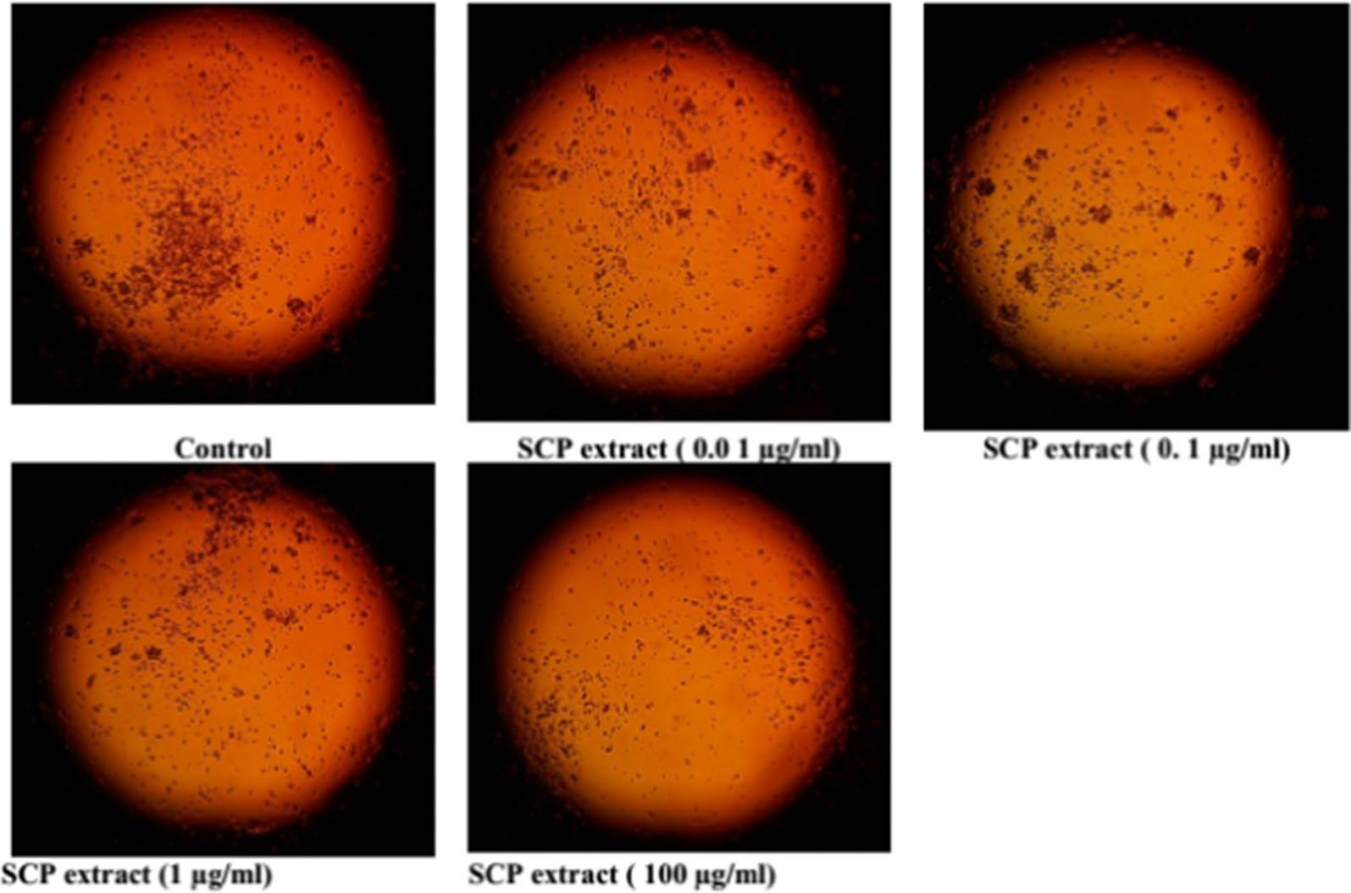
Photomicrographs of acute monocytic leukemia (THP1) cell lines for treatment with extract of SCP

The WST-1 assay was used to assess the cytotoxicity of plant extract on cell lines from patients with mononuclear acute myeloid leukemia (AML) and acute lymphocytic leukemia (ALL) after 24-h incubation. The findings showed that the 100% cell proliferation of the leukemic THP1 cells was significantly reduced in a dose-dependent manner to 97.25%, 88.33%, 86.49%, 76.69%, 72.06% upon treatment with 0.01, 0.1, 1, 10, and 100 µg/mL of SCP extract respectively. Accordingly, the obtained IC_50_ value was > 100 µg/ml, as shown in Figs. 8 and 9. The SCP extract was tested against lymphoma (U937) cells. The SCP extract reduced the viability of lymphoma U937 cells to 72.99%. See Figs. 8 and 10.

Acute lymphoblastic leukemia (ALL) malignancy causes lymphoid blast cells to accumulate in hematopoietic organs, particularly the bone marrow. The overproduction of damaged white blood cells causes leukemia. Although tyrosine kinase inhibitors are widely used, responses are phase-dependent, and recurrences are common. This research aimed to investigate the anticancer effect of the entire SCP extract against the THP1 leukemia cell line, which is extremely undifferentiated, and see if any apoptotic mechanisms are altered in vitro. Because synergetic effects could make combinatorial acts more noticeable, we decided to test the whole extract in leukemia cells rather than individual elements.

It is well-established that most phenolic compounds, glycosides, and tannins dissolve in ethanol and water solutions [61]. As a result, these chemical groups may include the most active compounds for leukemia [62]. Cell-to-cell communication is widely recognized as the most effective cell-type distinction in development and established tissue organization patterns [63]. At virtually every stage of development, cells send and receive signals from neighboring cells, which are essential for proper differentiation and function [64]. Numerous epidemiological studies have indicated an inverse correlation between a vegetable and fruit-rich diet and humans' risk of developing cancer [65]. Flavonoids and other phenolics are particularly intriguing candidates for preventing cancer [61]. There is significant evidence of the potential inhibitory effects of certain plant phenolic extracts on carcinogenesis and mutagenesis [63]. Due to the apparent diversity of dietary phenolics, including flavonoids, and the numerous possible pathways reported, scientists assumed that a single substance was not responsible for all the associations between cancer prevention and plant food intake [62]. El-Shemy et al. [62] suggested that phytochemicals in vegetables and fruits contributed significantly to their anticancer properties.

## Conclusions

The SCP extract contains a vast spectrum of bioactive substances. The antiviral activity of SCP extract against herpes simplex virus type II and adenovirus type 7 seems promising. The current study recommends this by-product for pharmaceutical applications and its antiviral, anticancer, and antioxidant properties. As a result, this extract should be advertised to the public as a health supplement due to its high concentration of antioxidants.

## Data Availability

The datasets utilized and analyzed during this investigation are available upon reasonable request from the corresponding author.
